# Association of Serum Calcium and Phosphate Concentrations with Glucose Metabolism Markers: The Furukawa Nutrition and Health Study

**DOI:** 10.3390/nu12082344

**Published:** 2020-08-05

**Authors:** Shamima Akter, Masafumi Eguchi, Takeshi Kochi, Isamu Kabe, Akiko Nanri, Tetsuya Mizoue

**Affiliations:** 1 Department of Epidemiology and Prevention, Center for Clinical Sciences, National Center for Global Health and Medicine, Tokyo 162-8655, Japan; nanri@fwu.ac.jp (A.N.); mizoue@hosp.ncgm.go.jp (T.M.); 2 Department of Health Administration, Furukawa Electric Corporation, Tokyo 100-8322, Japan; masafumi.eguchi@furukawaelectric.com (M.E.); takeshi.kochi@furukawaelectric.com (T.K.); isamu.kabe@furukawaelectric.com (I.K.); 3 Department of Food and Health Sciences, Fukuoka Women’s University, Fukuoka 813-8529, Japan

**Keywords:** calcium–phosphate product, insulin resistance, serum calcium, serum phosphate

## Abstract

Calcium and phosphate may play an important role in cardio-metabolic abnormalities, including type 2 diabetes; however, epidemiological evidence of the association of calcium and phosphate status with glucose metabolism among Asians is limited. In the current study, we performed a cross-sectional analysis of the association of serum calcium, phosphate, and calcium–phosphate product concentrations with glucose metabolism markers among Japanese individuals. Overall, 1701 workers (aged 18–78 years) who participated in a health survey were enrolled in this study. Multivariable linear regression models were used to estimate means of homeostatic model assessment of insulin resistance (HOMA-IR), homeostatic model assessment of β-cell function (HOMA-β), and glycated hemoglobin (HbA1c). Serum calcium concentration was positively associated with HOMA-IR and HbA1c (*p* for trend < 0.01). Multivariable-adjusted means (95% confidence interval (CI)) of HOMA-IR for the lowest and highest quartiles of serum calcium were 0.78 (0.75–0.82) and 1.01 (0.96–1.07), respectively. The corresponding values for HbA1c were 5.24 (5.22–5.27) and 5.29 (5.26–5.32), respectively. Serum phosphate and calcium–phosphate product concentrations were inversely associated with HOMA-IR (*p* for trend *<* 0.01). Multivariable-adjusted means (95% CI) of HOMA-IR for the lowest and highest quartiles of serum phosphate were 1.04 (0.99–1.09) and 0.72 (0.69–0.76), respectively. The corresponding values for calcium–phosphate product were 1.04 (0.99–1.09) and 0.73 (0.69–0.77), respectively. The current findings suggest that higher serum calcium and lower serum phosphate concentrations are associated with IR among apparently healthy adults.

## 1. Introduction

Besides other noncommunicable diseases, such as cardiovascular disease or cancer, diabetes mellitus is a major cause of death and disability [1,2]. The global prevalence of diabetes mellitus among adults has been reported to be approximately 9.3% in 2019 and is expected to further increase to 10.9% by 2045 [3]. Japan is one of the countries with the highest number of adults with diabetes, affecting approximately 7.4 million or 7.9% of the Japanese population [4]. Therefore, development of effective strategies is essential to combat this disease. In recent studies, attention has been paid to understand the role of calcium and phosphate in the etiology of type 2 diabetes (T2D). It has been suggested that T2D is associated with a common defect in calcium metabolism [5]. Several prospective studies have shown that higher circulating calcium levels are associated with an increased risk of T2D [6,7,8,9]. Elevated serum phosphate and calcium–phosphate product (calculated by multiplying calcium and phosphate concentrations) concentrations have also been reported to be associated with an increased risk of T2D [7]. The calcium–phosphate product is considered to be a clinically more relevant tool for estimating cardiometabolic risk among patients with chronic kidney disease than individual serum calcium or phosphate concentration [10]; however, evidence for this is limited to apparently healthy individuals, without chronic kidney disease. The mechanism underlying the associations among serum calcium, phosphate, and the calcium–phosphate product concentrations is not well-understood. Assessing insulin resistance (IR), an underlying condition of T2D, in relation to serum calcium and phosphate concentrations may provide important insight into this issue.

To date, few studies have investigated the association of serum calcium [7,11,12,13,14] and phosphate [7,14,15,16] concentrations with glucose metabolism markers, such as IR, fasting plasma glucose (FPG), or glycated hemoglobin (HbA1c). Among them, some [11,12,13,14], although not all [7], studies have reported that elevated calcium concentration is associated with increased FPG [11,12,14], IR [11,12,13], and HbA1c [11]. In contrast, a low phosphate concentration has been found to be associated with increased FPG [14,15] and insulin sensitivity [16]. In contrast, the calcium–phosphate product concentration was not found to be correlated with plasma glucose, insulin sensitivity, or insulin secretion [7]. However, limited evidence is available on these issues among Asian individuals, who have a lower body mass index (BMI) [17] and insulin secretion capacity [18] than Westerners. To the best of our knowledge, the association of serum calcium [13] and phosphate [15] concentrations with FPG and homeostatic model assessment of insulin resistance (HOMA-IR) has been investigated in only one Asian study previously. There has been no study of the association of serum phosphate with HbA1c levels which reflects a long-term glucose status.

To explore these issues further, the present study aimed to examine the association of circulating calcium, phosphate, and calcium–phosphate product concentrations with glucose metabolism markers, including fasting insulin, FPG, HOMA-IR, homeostatic model assessment of β-cell function (HOMA-β), and HbA1c levels among Japanese workers. We hypothesized that elevated blood calcium and decreased blood phosphate concentrations would be associated with dysregulated glucose metabolism markers in this apparently healthy population.

## 2. Materials and Methods

### 2.1. Study Procedures

Data for the present study were derived from the Furukawa Nutrition and Health Study, the details of which have been described previously [19]. At the time of a periodic health checkup, all the workers (white-collar (58%) or blue-collar (42%)) from two sites of a manufacturing company in Japan were invited to participate in the survey. In brief, the survey was conducted at baseline (in April 2012 and May 2013) and in a 3-year follow-up session (in April 2015 and May 2016). The present study was conducted according to the 3-year follow-up survey (second survey), when serum calcium and phosphorous concentrations were measured. During the second survey, among 2350 eligible participants, 2067 Japanese employees participated in the survey (response rate: 88%). On the day of the health checkup, research staff checked the questionnaire for completeness and clarified responses with the participants, where necessary. Anthropometric measurements were performed, and venous blood was collected at the workplace, during the routine health checkup. Health checkup data were obtained, including the results of anthropometric and biochemical measurements and disease history. All subjects provided informed consent for inclusion before they participated in the study. The study was conducted in accordance with the Declaration of Helsinki, and the protocol was approved by the Ethics Committee of the National Center for Global Health and Medicine, Japan (ethical approval number NCGM-G-001140-15).

### 2.2. Assessment of Dietary Intakes

Dietary habits during the preceding 1-month period were assessed by using a validated brief self-administered questionnaire (BDHQ). Dietary intakes of 58 food and beverage items and levels of energy and selected nutrients, such as calcium, vitamin D, and phosphorous, were estimated by using an ad hoc computer algorithm for the BDHQ, according to the Standard Tables of Food Composition in Japan [20]. 

### 2.3. Blood Measurements

Blood samples were obtained during the health examinations in 2015–2016. Venous blood (7 mL) donated for the study was drawn into a vacuum tube and was then taken in a cooler box to a laboratory. Blood was centrifuged for 15 min, to separate the serum, and the serum sample was stored at −80 °C, until the analyses were performed. As part of the health checkup, FPG concentrations were assayed enzymatically, using Quick-auto-neo-GLU-HK and Quick-auto-II-GLU-HK (Shino-Test Corp., Tokyo, Japan), and HbA1c levels were measured by latex agglutination immunoassay, using the Determiner HbA1c and Determiner L HbA1c kits (Kyowa Medex Co., Ltd., Tokyo, Japan), at an external laboratory (Kinki Kenko Kanri Center, Shiga, Japan). Serum calcium, phosphate, and insulin concentrations were measured at an external laboratory (Mitsubishi Chemical Medience Corporation, Tokyo, Japan). Serum calcium and phosphate concentrations were measured by using an Arsenazo III dye method [21] and an enzymatic method [22], respectively. Calcium–phosphate product concentration was calculated as calcium concentration × phosphate concentration. Serum insulin levels were measured by using a chemiluminescence immunoassay [23], with intra-assay coefficients of variation of 2.5% at 43.1 pM and 1.2% at 423 pM. We computed HOMA-IR and HOMA-β scores, using the following formulas: HOMA-IR = (fasting insulin (μU/mL) × fasting glucose (mg/dL))/405 [24] and HOMA-β = 360 × (fasting insulin/(fasting glucose − 63)) [24].

### 2.4. Assessment of Other Health-Related Parameters

Body height and weight were measured to the nearest 0.1 cm and 0.1 kg, respectively, with participants wearing the least amount of clothing and without shoes. BMI was calculated by using the following formula: kg/m^2^, where kg refers to the person’s weight in kilograms and m^2^ to their height in meters squared. Smoking status, alcohol consumption, night and rotating shift work, work-related activities, and leisure-time activities were self-reported, using the lifestyle questionnaire. The average daily alcohol intake was calculated as the frequency of drinking alcoholic beverages multiplied by alcohol consumption per drinking day. Total alcohol consumption was reported in *go* (180 mL), the conventional unit for measuring alcohol in Japan. Work-related and leisure-time physical activities were each expressed as the sum of their metabolic equivalent (MET) values multiplied by the duration of that activity.

### 2.5. Participants

**Figure 1:**
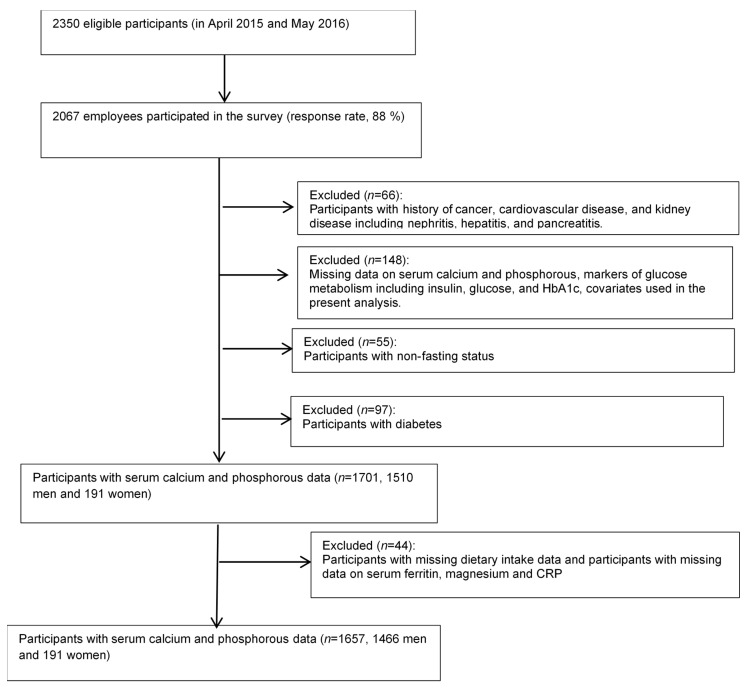
Flowchart of the study population.

Among 2067 participants who responded to the second survey, we excluded 66 participants with a history of cancer (*n* = 27), cardiovascular disease (*n* = 30), or kidney disease, including nephritis (*n* = 8), hepatitis (*n* = 2), and pancreatitis (*n* = 2) (Figure 1). Some participants had two or more conditions for exclusion. Of the remaining participants, we then excluded 148 participants who had missing data on serum calcium and phosphorous concentrations (*n* = 131); glucose metabolism markers, such as insulin, glucose, or HbA1c (*n* = 5); covariates used in the main analysis (*n* = 12); and a non-fasting status at the time of blood sampling (*n* = 55). We further excluded 97 participants with diabetes (defined as an FPG ≥ 126 mg/dL; HbA1c ≥ 6.5% (≥48 mmol/mol) or who were under medical treatment for diabetes), leaving 1701 participants (1510 men and 191 women) for the main analysis. We further excluded nine and 35 participants who had missing data on dietary intake and serum ferritin, magnesium, and C-reactive protein (CRP), respectively, leaving 1657 participants (1466 men and 191 women) for additional analyses.

### 2.6. Statistical Analyses

General characteristics of the study population are presented as proportions and means according to quartiles of serum calcium and phosphate. Associations of potential confounding variables with serum calcium and phosphate concentrations were examined by using the generalized linear regression model, by considering the median value of each quartile of the respective categories and modeling this as a continuous variable. Spearman correlation coefficients were calculated to assess the correlation between dietary and serum calcium and phosphate concentrations.

Because of the skewed distribution of fasting insulin, glucose, HOMA-IR, and HOMA-β, these values were log-transformed, to achieve a better approximation of a normal distribution before analyses were conducted. Multiple linear regression analysis was used to calculate adjusted geometric means (95% confidence interval (CI)) for fasting levels of insulin, glucose, and HOMA-IR, and arithmetic means (95% CIs) for HbA1c levels according to the quartiles of serum calcium, phosphate, and calcium–phosphate product. We adjusted for age (year, continuous), sex, and work (site A or site B) in the first model. We further adjusted for work-related physical activity (<3, 3 to <7, 7 to <20, or ≥ 20 METs-h/day), leisure-time physical activity (0, 0.1 to <3, 3 to <10, or ≥10 METs-h/week), smoking status (never-smoker, quitter, current smoker consuming < 20 cigarettes/day, or current smoker consuming ≥ 20 cigarettes/day), alcohol consumption (non-drinker, including infrequent drinker consuming less than one drink per week, drinkers consuming < 23 g of ethanol/day, drinkers consuming ≥ 23 to <46 g of ethanol/day, and drinkers consuming ≥ 46 g of ethanol/day), night or rotating shift work (yes or no), and BMI (kg/m^2^, continuous) in the second model. To assess whether serum calcium and phosphate concentrations were associated with glucose metabolism markers independently of other specific nutrient intakes and risk factors that have been associated with glucose metabolism markers in previous studies [19,25,26,27,28], we further adjusted for logarithmic CRP (mg/dL), serum magnesium (mg/dL), serum ferritin (ng/mL), dietary calcium intake (mg/1000 kcal/day, continuous), and vitamin D intake (mg/1000 kcal/day, continuous) in the third model. Trends for associations of serum calcium, phosphate, and calcium–phosphate product concentrations with glucose metabolism markers were determined by assigning the median value of each exposure quartile to the appropriate category and modeling it as a continuous variable. We repeated the above analysis, considering serum calcium, phosphate, and the calcium–phosphate product concentrations as continuous variables. Two-sided *p-*values < 0.05 were considered statistically significant. All analyses were performed by using the statistical software Stata version 15.1 (StataCorp, College Station, TX, USA).

## 3. Results

Table 1 shows the characteristics of study participants who participated in the 2015–2016 survey, across quartiles of serum calcium and phosphate. Participants with higher serum calcium levels were younger, mostly men, and alcohol drinkers. Serum calcium levels were positively associated with serum ferritin and vitamin D intake. Participants with higher serum phosphate levels were younger, mostly shift workers, and had higher serum magnesium concentrations.

There was no correlation between serum calcium concentration and dietary calcium intake or serum phosphate concentration and dietary phosphorous intake (Spearman’s correlation coefficient = 0.046 and 0.023; *p* = 0.06 and 0.34 for calcium and phosphorous, respectively). Furthermore, no correlation was observed between serum calcium and serum phosphate concentrations (Spearman’s correlation coefficient = 0.04; *p* = 0.12). The calcium–phosphate product concentration was highly correlated with serum phosphate concentration (Spearman’s correlation coefficient = 0.99; *p* < 0.001); however, it was weakly correlated with serum calcium concentration (Spearman’s correlation coefficient = 0.15; *p* < 0.001).

Serum calcium concentration was positively associated with fasting insulin, FPG, HOMA-IR, HOMA-β, and HbA1c, among all the models (Table 2). The means of fasting insulin (μU/mL) (95% CI) for the lowest through highest quartiles of serum calcium, according to the multivariable model adjusted for age, sex, work, work-related physical activity, leisure-time physical activity, smoking, alcohol drinking, night or rotating shift work, and BMI (model 2), were 3.59 (3.44–4.74), 3.84 (3.67–4.01), 4.01 (3.81–4.22), and 4.48 (4.24–4.73) (*p* for trend < 0.001), respectively. The corresponding values of FPG (mg/dL) were 89.3 (88.6–90.1), 90.1 (89.3–90.9), 90.1 (89.2–91.0), and 92.2 (91.2–93.2), respectively (*p* for trend < 0.001). The corresponding values of HOMA-IR were 0.79 (0.76–0.83), 0.85 (0.81–0.89), 0.89 (0.85–0.94), and 1.02 (0.96–1.08), respectively (*p* for trend < 0.01). The corresponding values of HOMA-β were 51.2 (49.1–53.5), 53.5 (50.9–55.9), 54.9 (52.1–57.9), and 57.9 (54.7–61.2), respectively (*p* for trend < 0.01). The corresponding values of HbA1c (%) were 5.25 (5.22–5.27), 5.30 (5.27–5.32), 5.30 (5.27–5.33), and 5.28 (5.25–5.31), respectively (*p* for trend = 0.02). These associations remained virtually unchanged after further adjustment for serum calcium, magnesium, ferritin, and CRP concentrations and dietary calcium and vitamin D intakes (model 3). Serum calcium concentration on a continuous scale was also significantly and positively associated with fasting insulin, FPG, HOMA-IR, HOMA-β, and HbA1c levels (*p* for trend < 0.05 for all).

Serum phosphate concentration was significantly and inversely associated with fasting insulin, FPG, HOMA-IR, and HOMA-β among all the models (Table 3). The adjusted means (95% CI) of fasting insulin (μU/mL) in model 2, for the lowest to the highest quartiles of serum phosphate, were 4.52 (4.32–4.74), 4.03 (3.85–4.23), 3.73 (3.57–3.91), and 3.37 (3.21–3.54), respectively, *p* for trend < 0.01). The corresponding values for fasting glucose (mg/dL) were 92.9 (92.0–93.8), 90.5 (89.7–91.4), 89.8 (89.0–90.7), and 87.5 (86.7–88.4), respectively (*p* for trend < 0.01). The corresponding values of HOMA-IR were 1.03 (0.99–1.09), 0.90 (0.86–0.95), 0.83 (0.79–0.87), and 0.73 (0.69–0.77), respectively (*p* for trend < 0.01). Serum phosphate concentration were not associated with HbA1c. Similar associations were found in model 3 after further adjustments for serum phosphate, serum magnesium, serum ferritin, CRP, and dietary calcium and vitamin D intakes. In the adjusted model, which included serum phosphate concentration as a continuous variable, serum phosphate concentration was inversely associated with fasting insulin, fasting glucose, HOMA-IR, and HOMA-β (*p* < 0.05 for all). Serum phosphate concentration on a continuous scale was also significantly and inversely associated with fasting insulin, FPG, HOMA-IR, and HOMA-β (*p* for trend < 0.05 for all).

Table 4 shows the geometric means of glucose metabolism markers, including fasting insulin, glucose, HOMA-IR, and HOMA-β, and means of HbA1c, according to the quartiles of the calcium–phosphate product. The serum calcium–phosphate product concentration was significantly and inversely associated with fasting insulin, fasting glucose, HOMA-IR, and HOMA-β, which is similar to that of serum phosphate concentration.

## 4. Discussion

In the present cross-sectional study, serum calcium concentrations were significantly and positively associated with glucose metabolism markers, including fasting insulin, FPG, HOMA-IR, HOMA-β, and HbA1c. In contrast, serum phosphate and calcium–phosphate product concentrations were significantly and inversely associated with all glucose metabolism markers, except HbA1c. To the best of our knowledge, few studies have, to date, investigated the association of serum calcium and phosphate concentrations with glucose metabolism markers.

The positive association of serum calcium concentration with fasting insulin, FPG, and HOMA-IR we observed in our Japanese study population was consistent with the results of previous studies among Westerners [11,12,14] and Asians [13]. In an Indian study among 2699 individuals (aged 40–84 years), serum calcium concentration was positively associated with fasting insulin and HOMA-IR [13]. In a Canadian study among 1182 adults without diabetes, serum calcium concentration was positively correlated with FPG and HOMA-IR [12]. In two large-scale US studies among community adults [11,14], serum calcium concentration was positively associated with FPG [14], 2-h glucose [11], and fasting insulin [11]. Altogether, the results of the present study suggest that high serum calcium concentration is associated with increased fasting insulin, FPG, and HOMA-IR, which are markers of IR.

In the present study, we found a positive association between serum calcium concentration and HOMA-β. In a Canadian study, a significant inverse correlation was found between serum calcium concentrations and HOMA-β among women, although not among men [12]. In a Swedish study, among 961 elderly individuals without diabetes, serum calcium concentration was not associated with insulin secretion measured as the early insulin response during an oral glucose tolerance test [29]. The reasons for the inconsistencies between the present and previous studies are not clear; however, they might be because of different ethnic and background characteristics of the study populations. The present findings can mechanistically be explained by the fact that when blood sugar levels rise among healthy individuals, it is efficiently taken up by beta cells and IR results in a compensatory increase in insulin secretion, leading to the occurrence of defective and decreased insulin secretion [30]. Because of the limited epidemiologic data, further studies are necessary to elucidate the role of calcium in insulin secretion.

To date, no study has been conducted to assess the association between serum phosphate concentration and HOMA-IR. The observed inverse association of serum phosphate concentration with FPG is in agreement with the findings of previous studies [14,15,16]. In a large Japanese study conducted among 16,041 individuals undergoing routine health checkups at the Iida Municipal Hospital [15], serum phosphate concentration was significantly and inversely associated with FPG. In a large-scale community-based study (*n* = 15732), the Atherosclerosis Risk in Communities Study [14], serum phosphate concentration was also inversely associated with FPG. In a German study, among 881 participants without diabetes having a family history of T2D [16], an inverse association was found between phosphate concentration and two-hour blood glucose concentration. Altogether, serum phosphate concentration might be inversely associated with IR among apparently healthy individuals.

Unlike HOMA-IR, the associations of both serum calcium and phosphate concentrations with HbA1c in the present study appeared to be much weaker and non-linear. This finding may be partially attributed to the fact that this study only included healthy participants without diabetes who perhaps had highly functional beta-cells capable of secreting a sufficient amount of insulin to control their blood glucose levels, even in the presence of IR.

In the present study, we found an inverse association of calcium–phosphate product concentration with fasting insulin, FPG, and HOMA-IR, which is similar to that of serum phosphate concentration. Given the very high correlation between serum phosphate and the calcium–phosphate product concentrations, the association of the calcium–phosphate product concentration with IR was likely driven by the association of serum phosphate concentration with IR. A US study found no correlation of the calcium–phosphate product concentration with FPG, insulin sensitivity, and insulin secretion [7]. Likewise, in another US study, no significant association was found between the calcium–phosphate product concentration and FPG [14]. Further studies are necessary to elucidate the role of the calcium–phosphate product in glucose metabolism among individuals without renal failure.

The underlying mechanism by which high calcium and low phosphate concentrations increased IR remains unclear, although there are some possible explanations. Higher intracellular calcium levels may cause a decline in the effect of insulin in adipocytes by decreasing the number of glucose transporters, particularly glucose transporter type 4 and insulin receptor activity [31,32]. Elevated calcium concentration may lead to inflammation [33], and, according to experimental studies, proinflammatory cytokines, such as tumor necrosis factor-α (TNF-α) could cause IR [34]. However, phosphate is involved in energy balance and adenosine triphosphate generation [35,36], and reduction of serum phosphate concentration could lead to disturbances in energy metabolism, resulting in IR [37]. Although the mechanism underlying the positive association between serum calcium concentration and HOMA-β is unclear, there are some possible explanations. Systemic calcium (Ca^2+^) signaling, including intracellular Ca^2+^ levels, Ca^2+^ dependent enzymes, and Ca^2+^ channels, is one of the key factors that controls insulin secretion in beta cells [38]. In experimental studies, the calcium-sensing receptor, which controls calcium homeostasis as an extracellular receptor, is expressed in the beta cells [39], and its activation distinctly increases the insulin secretory responses [40,41].

The major strengths of the present study include the high study participation rate, inclusion of a variety of glucose metabolism markers, and consideration of several potential confounding variables. However, there are some limitations of this study that need to be mentioned. First, an association observed from a cross-sectional study does not necessarily indicate causality. Second, we did not use a glucose clamp method, which is considered a gold-standard technique for measuring IR. However, in the present study, we used the HOMA model, which is a robust and widely applied method for estimating IR [42]. Third, total blood calcium levels vary with serum albumin levels because of calcium–albumin binding [43], and albumin-corrected calcium may provide a true association between serum calcium concentration and glucose metabolism markers. However, no data were available on the blood albumin levels; therefore, it was not possible to estimate albumin-corrected calcium concentration. Fourth, we cannot rule out the possibility of bias because of residual confounding and unmeasured factors. Finally, the results obtained in this study may not apply to the general population, because the study was conducted only in a selected workplace.

In conclusion, higher serum calcium and lower serum phosphate and calcium–phosphate product concentrations are associated with higher fasting insulin, FPG, HOMA-IR, and HOMA-β concentrations among apparently healthy Japanese adults. Higher serum calcium concentration are also associated with higher HbA1c concentration. Further prospective studies are necessary to confirm the present findings.

## Figures and Tables

**Table 1:** Characteristics of the study participants by quartiles of serum calcium and phosphate.

	Quartiles of Serum Calcium (mg/dL)					Quartiles of Serum Phosphate (mg/dL)				
	Q_1_ (Low)	Q_2_	Q_3_	Q_4_ (High)	Trend *p* ^a^	Q_1_ (Low)	Q_2_	Q_3_	Q_4_ (High)	Trend *p* ^a^
Number of participants	574	462	371	324		443	423	452	413	
Median	9.3	9.6	9.7	10.0		2.6	3.1	3.7	4.7	
Range	8.6–9.4	9.5–9.6	9.7–9.8	9.9–11.1		1.4–2.8	2.9–3.3	3.4–4.1	4.2–7.3	
Age (years)	45.7 ± 8.3	44.7 ± 8.5	44.8 ± 8.8	43.2 ± 10.4	<0.01	47.1 ± 9.2	44.9 ± 9.4	43.8 ± 8.6	43.2 ± 7.5	<0.01
Sex (men, %)	84.5	89.6	89.5	95.1	<0.01	91.2	84.6	90.9	88.6	0.88
Leisure-time physical activity (METs-h/week)	8.4 ± 15.9	8.9 ± 18.2	9.3 ± 19.4	9.3 ± 19.0	0.44	7.7 ± 13.6	9.1 ± 17.6	10.6 ± 22.8	8.1 ± 16.0	0.30
Work-related physical activity (METs-h/day)	15.9 ± 18.0	14.8 ± 16.8	15.5 ± 17.9	13.8 ± 15.3	0.10	15.3 ± 16.9	15.4 ± 18.4	15.0 ± 17.4	15.0 ± 16.0	0.67
BMI (kg/m^2^)	23.6 ± 3.4	23.6 ± 3.4	23.8 ± 3.6	23.6 ± 3.6	0.92	24.0 ± 3.8	23.4 ± 3.4	23.5 ± 3.3	23.6 ± 3.5	0.28
Current smoker (%)	31.7	29.4	28.6	27.2	0.13	27.3	26.5	32.7	31.7	0.06
Current alcohol drinker (≥1 day/week, %)	49.8	56.1	56.3	55.6	0.05	54.2	52.7	52.0	57.1	0.34
Night and rotating shift work (yes, %)	18.3	18.2	19.9	16.7	0.71	17.2	14.9	19.9	21.3	0.03
CRP (mg/dL)	0.09 ± 0.26	0.09 ± 0.29	0.07 ± 0.18	0.08 ± 0.15	0.25	0.09 ± 0.27	0.07 ± 0.20	0.08 ± 0.22	0.09 ± 0.24	0.60
Serum ferritin (ng/mL)	145 ± 114	165 ± 122	169 ± 113	204 ± 174	<0.01	180 ± 163	155 ± 104	163 ± 119	169 ± 131	0.67
Serum magnesium (mg/dL)	2.21 ± 0.14	2.20 ± 0.14	2.23 ± 0.14	2.22 ± 0.14	0.20	2.19 ± 0.14	2.21 ± 0.14	2.23 ± 0.14	2.24 ± 0.14	<0.01
Calcium intake (mg/1000 kcal/day)	235 ± 95	239 ± 91	239 ± 90	236 ± 89	0.70	238 ± 92	243 ± 97	236 ± 87	232 ± 90	0.19
Phosphorous intake (mg/1000 kcal/day)	507 ± 102	516 ± 110	519 ± 102	512 ± 102	0.26	514 ± 98	519 ± 110	514 ± 100	505 ± 106	0.08
Vitamin D intake (mg/1000 kcal/day)	5.7 ± 3.2	6.2 ± 3.8	6.3 ± 3.9	6.1 ± 3.4	0.04	6.2 ± 3.5	6.3 ± 3.8	6.0 ± 3.4	5.8 ± 3.6	0.09

Abbreviations: METs, metabolic equivalents; BMI, body mass index; CRP, C-reactive protein. Data are presented as the means ± standard deviations or as percentages. ^a^ Linear trends across quartiles of serum calcium and phosphate were tested by entering the median value of each quartile into the generalized linear model.

**Table 2:** Adjusted means (95% CI) of insulin, glucose, HOMA-IR, HOMA-β, and HbA1c across quartiles of serum calcium.

	Quartiles of Serum Calcium (mg/dL)				Trend *p* ^a^
	Q_1_ (Low)	Q_2_	Q_3_	Q_4_ (High)	
Number of subjects	574	462	371	324	
Median (range)	9.3 (8.6–9.4)	9.6 (9.5–9.6)	9.7 (9.7–9.8)	10.0 (9.9–11.1)	
**Fasting insulin (μU/mL)**					
Model 1 ^b^	3.62 (3.45–3.80)	3.80 (3.60–4.01)	4.06 (3.83–4.31)	4.41 (4.14–4.70)	<0.001
Model 2 ^c^	3.59 (3.44–3.74)	3.84 (3.67–4.01)	4.01 (3.81–4.22)	4.48 (4.24–4.73)	<0.001
Model 3 ^d^	3.58 (3.44–3.72)	3.90 (3.74–4.08)	4.04 (3.85–4.24)	4.50 (4.27–4.74)	<0.001
**Fasting glucose (mg/dL**)					
Model 1 ^b^	89.3 (88.5–90.1)	90.1 (89.2–90.9)	90.2 (89.3–91.2)	92.1 (91.0–93.1)	<0.001
Model 2 ^c^	89.3 (88.6–90.1)	90.1 (89.3–90.9)	90.1 (89.2–91.0)	92.2 (91.2–93.2)	<0.001
Model 3 ^d^	88.6 (88.0–89.3)	89.4 (88.7–90.1)	89.8 (89.1–90.6)	91.4 (90.5–92.3)	<0.001
**HOMA-IR**					
Model 1 ^b^	0.80 (0.76–0.84)	0.84 (0.80–0.89)	0.90 (0.85–0.96)	1.00 (0.94–1.07)	<0.001
Model 2 ^c^	0.79 (0.76–0.83)	0.85 (0.81–0.89)	0.89 (0.85–0.94)	1.02 (0.96–1.08)	<0.001
Model 3 ^d^	0.78 (0.75–0.82)	0.86 (0.82–0.90)	0.90 (0.85–0.94)	1.01 (0.96–1.07)	<0.01
**HOMA-β**					
Model 1 ^b^	51.7 (49.4–54.2)	52.9 (50.2–55.7)	55.2 (52.1–58.6)	57.2 (53.7–60.9)	0.01
Model 2 ^c^	51.2 (49.1–53.5)	53.3 (50.9–55.9)	54.9 (52.1–57.9)	57.9 (54.7–61.2)	0.001
Model 3 ^d^	52.1 (50.1–54.3)	55.1 (52.6–57.6)	56.2 (53.4–59.1)	59.4 (56.3–62.7)	<0.001
**HbA1c (%)**					
Model 1 ^b^	5.25 (5.23–5.27)	5.29 (5.27–5.32)	5.30 (5.28–5.33)	5.28 (5.25–5.31)	0.057
Model 2 ^c^	5.25 (5.22–5.27)	5.30 (5.27–5.32)	5.30 (5.27–5.33)	5.28 (5.25–5.31)	0.02
Model 3 ^d^	5.24 (5.22–5.27)	5.30 (5.27–5.32)	5.30 (5.28–5.33)	5.29 (5.26–5.32)	0.01

Abbreviations: HOMA-IR, homeostatic model assessment of insulin resistance; HOMA-β, homeostatic model assessment of β-cell function; HbA1c, glycated hemoglobin. ^a^ Linear trends across quartiles of serum calcium level were tested by entering the median values of each category into the generalized linear model. ^b^ Model 1 adjusted for age (year, continuous), sex, and site (A or B). ^c^ Model 2 additionally adjusted for smoking (never-smoker, quitter, current smoker consuming < 20 cigarettes/day, or current smoker consuming ≥ 20 cigarettes/day), alcohol drinking (non-drinker, occasional drinker, or drinker consuming < 23 g of ethanol/day, drinker consuming 23–45 g of ethanol/day, or drinker consuming ≥ 46 g of ethanol/day), work-related physical activity (metabolic equivalents (METs)—h/day, quartile), leisure-time physical activity (METs—h/week, quartile), night or rotating shift work (yes or no), body mass index (kg/m^2^, continuous). ^d^ Model 3 additionally adjusted for serum phosphate, serum magnesium, serum ferritin, log transformed C-reactive protein (mg/dL), dietary calcium (mg/1000 kcal/day) and vitamin D (mg/1000 kcal/day) intakes.

**Table 3:** Adjusted means (95% CI) of insulin, glucose, HOMA-IR, HOMA-β, and HbA1c across quartiles of serum phosphate.

	Quartiles of Serum Phosphate (mg/dL)				Trend *p* ^a^
	Q_1_ (Low)	Q_2_	Q_3_	Q_4_ (High)	
Number of subjects	443	423	452	413	
Median (range)	2.6 (1.4–2.8)	3.1 (2.9–3.3)	3.7 (3.4–4.1)	4.7 (4.2–7.3)	
**Fasting insulin (μU/mL)**					
Model 1 ^b^	4.67 (4.42–4.93)	3.97 (3.76–4.20)	3.70 (3.50–3.90)	3.35 (3.17–3.54)	<0.001
Model 2 ^c^	4.52 (4.32–4.74)	4.03 (3.85–4.23)	3.73 (3.57–3.91)	3.37 (3.21–3.54)	<0.001
Model 3 ^d^	4.59 (4.39–4.80)	4.14 (3.97–4.32)	3.66 (3.49–3.83)	3.35 (3.20–3.51)	<0.001
**Fasting glucose (mg/dL**)					
Model 1 ^b^	93.1 (92.2–94.0)	90.3 (89.4–91.2)	89.7 (88.9–90.6)	87.7 (86.8–88.6)	<0.001
Model 2 ^c^	92.9 (92.0–93.8)	90.5 (89.7–91.4)	89.8 (89.0–90.7)	87.5 (86.7–88.4)	<0.001
Model 3 ^d^	91.8 (91.1–92.6)	89.5 (88.9–92.6)	89.4 (88.7–90.2)	87.6 (86.9–88.3)	<0.001
**HOMA-IR**					
Model 1 ^b^	1.07 (1.01–1.14)	0.88 (0.83–0.94)	0.82 (0.77–0.87)	0.72 (0.68–0.77)	<0.001
Model 2 ^c^	1.03 (0.99–1.09)	0.90 (0.86–0.95)	0.83 (0.79–0.87)	0.73 (0.69–0.77)	<0.001
Model 3 ^d^	1.04 (0.99–1.09)	0.92 (0.88–0.96)	0.81 (0.77–0.85)	0.72 (0.69–0.76)	<0.001
**HOMA-β**					
Model 1 ^b^	54.0 (52.6–55.5)	53.6 (52.2–55.1)	53.6 (52.2–55.0)	53.8 (52.4–55.3)	<0.001
Model 2 ^c^	57.2 (54.5–60.1)	55.4 (52.7–58.2)	51.7 (49.3–54.3)	50.8 (48.3–53.5)	<0.001
Model 3 ^d^	59.5 (56.8–62.4)	58.2 (55.7–60.9)	51.5 (49.0–54.0)	50.9 (48.5–53.4)	<0.001
**HbA1c (%)**					
Model 1 ^b^	5.29 (5.26–5.31)	5.28 (5.25-5.30)	5.31 (5.29–5.34)	5.25 (5.22–5.27)	0.07
Model 2 ^c^	5.28 (5.25–5.30)	5.28 (5.26–5.31)	5.31 (5.28–5.33)	5.25 (5.22–5.27)	0.11
Model 3 ^d^	5.28 (5.25–5.30)	5.29 (5.26–5.31)	5.30 (5.28–5.33)	5.25 (5.22–5.27)	0.08

Abbreviations: HOMA-IR, homeostatic model assessment of insulin resistance; HOMA-β, homeostatic model assessment of β-cell function; HbA1c, glycated hemoglobin. ^a^ Linear trends across quartiles of serum phosphate were tested by entering the median values of each category into the generalized linear model. ^b^ Model 1 adjusted for age (year, continuous), sex, and site (A or B). ^c^ Model 2 additionally adjusted for smoking (never-smoker, quitter, current smoker consuming < 20 cigarettes/day, or current smoker consuming ≥ 20 cigarettes/day), alcohol drinking (non-drinker, occasional drinker, or drinker consuming < 23 g of ethanol/day, drinker consuming 23–45 g of ethanol/day, or drinker consuming ≥ 46 g of ethanol/day), work-related physical activity (metabolic equivalents (METs)—h/day, quartile), leisure-time physical activity (METs—h/week, quartile), night or rotating shift work (yes or no), body mass index (kg/m^2^, continuous). ^d^ Model 3 additionally adjusted for serum calcium, serum magnesium, serum ferritin, log transformed C-reactive protein (mg/dL), dietary calcium (mg/1000 kcal/day), and vitamin D (mg/1000 kcal/day) intakes.

**Table 4:** Adjusted means (95% CI) of insulin, glucose, HOMA-IR, HOMA-β, and HbA1c across quartiles of serum-calcium-and-phosphate product.

	Quartiles of Serum-Calcium-and-Phosphate Product				Trend *p* ^a^
	Q_1_ (Low)	Q_2_	Q_3_	Q_4_ (High)	
Number of subjects	432	424	422	423	
Median (range)	24.4 (13.7–27.3)	29.7 (27.3–32.2)	35.4 (32.3–39.6)	45.1 (39.6–73.7)	
**Fasting insulin (μU/mL)**					
Model 1 ^b^	4.52 (4.28–4.77)	3.93 (3.72–4.15)	3.62 (3.43–3.82)	3.45 (3.27–3.65)	<0.001
Model 2 ^c^	4.42 (4.22–4.63)	4.01 (3.82–4.20)	3.67 (3.50–3.85)	3.41 (3.26–3.58)	<0.001
Model 3 ^d^	4.61 (4.40–4.82)	4.09 (3.91–4.28)	3.71 (3.55–3.88)	3.38 (3.23–3.54)	<0.001
**Fasting glucose (mg/dL)**					
Model 1 ^b^	91.4 (90.6–92.1)	89.5 (88.7–90.2)	89.5 (88.8–90.3)	87.8 (87.1–88.6)	<0.001
Model 2 ^c^	91.3 (90.5–92.0)	89.7 (88.9–90.4)	89.7 (88.9–90.4)	87.6 (86.9–88.4)	<0.001
Model 3 ^d^	91.6 (90.9–92.4)	89.7 (88.9–90.4)	89.6 (88.9–90.4)	87.5 (86.8–88.2)	<0.001
**HOMA-IR**					
Model 1 ^b^	1.02 (0.96–1.08)	0.87 (0.82–0.92)	0.80 (0.75–0.85)	0.75 (0.71–0.79)	<0.001
Model 2 ^c^	1.00 (0.95–1.05)	0.89 (0.84–0.93)	0.81 (0.77–0.85)	0.74 (0.70–0.78)	<0.001
Model 3 ^d^	1.04 (0.99–1.09)	0.91 (0.86–0.95)	0.82 (0.78–0.86)	0.73 (0.69–0.77)	<0.001
**HOMA-β**					
Model 1 ^b^	59.4 (56.3–62.7)	55.4 (52.5–58.5)	50.7 (48.1–53.5)	52.1 (49.3–54.9)	<0.001
Model 2 ^c^	58.4 (55.6–61.2)	56.2 (53.5–58.9)	51.2 (48.8–53.7)	51.8 (49.4–54.4)	<0.001
Model 3 ^d^	59.6 (56.8–62.5)	57.4 (54.8–60.2)	51.8 (49.4–54.2)	52.1 (49.7–54.6)	<0.001
**HbA1c (%)**					
Model 1 ^b^	5.29 (5.26–5.31)	5.27 (5.24–5.30)	5.31 (5.28–5.33)	5.26 (5.23–5.28)	0.38
Model 2 ^c^	5.27 (5.25–5.30)	5.28 (5.25–5.30)	5.31 (5.29–5.34)	5.26 (5.23–5.28)	0.54
Model 3 ^d^	5.27 (5.24–5.30)	5.28 (5.26–5.31)	5.31 (5.28–5.33)	5.25 (5.22–5.28)	0.43

Abbreviations: HOMA-IR, homeostatic model assessment of insulin resistance; HOMA-β, homeostatic model assessment of β-cell function; HbA1c, glycated hemoglobin. ^a^ Linear trends across quartiles of serum phosphate were tested by entering the median values of each category into the generalized linear model. ^b^ Model 1 adjusted for age (year, continuous), sex, and site (A or B). ^c^ Model 2 additionally adjusted for smoking (never-smoker, quitter, current smoker consuming < 20 cigarettes/day, or current smoker consuming ≥ 20 cigarettes/day), alcohol drinking (non-drinker, occasional drinker, or drinker consuming < 23 g of ethanol/day, drinker consuming 23–45 g of ethanol/day, or drinker consuming ≥ 46 g of ethanol/day), work-related physical activity (metabolic equivalents (METs)—h/day, quartile), leisure-time physical activity (METs—h/week, quartile), night or rotating shift work (yes or no), body mass index (kg/m^2^, continuous). ^d^ Model 3 additionally adjusted serum magnesium, serum ferritin, and log transformed C-reactive protein (mg/dL). A similar association was found when we examined the changes in glucose metabolism markers per one standard deviation change in serum calcium, phosphate, and calcium–phosphate product concentrations as continuous variables (Supplementary Materials Table S1).

## Supplementary Materials

The following are available online at https://www.mdpi.com/2072-6643/12/8/2344/s1. Table S1: Changes in glucose metabolism markers per 1 SD change in serum calcium and phosphate levels and calcium-phosphate products.
