# Supplementary material for: Association of Serum Calcium and Phosphate Concentrations with Glucose Metabolism Markers: The Furukawa Nutrition and Health Study
Source: Nutrients. 2020 Aug 5;12(8):2344. doi: 10.3390/nu12082344 (PMC7468836; doi:10.3390/nu12082344)
Supplement: Supplementary file 1 [file nutrients-12-02344-s001.pdf]

Supplemental Table 1 Changes in glucose metabolism markers per 1 SD change in serum calcium and phosphate levels and calcium-phosphate products.

	Calcium	Phosphate	Calcium-phosphate product
**Fasting insulin (μU/mL)**			
Model 1[a]	0.25*	- 0.47*	- 0.44*
Model 2[b]	0.24*	- 0.49*	- 0.46*
**Fasting glucose (mg/dL)**			
Model 1[a]	1.01*	- 1.57*	- 1.44*
Model 2[b]	1.08*	- 1.64*	- 1.46*
**HOMA-IR**			
Model 1[a]	0.07*	- 0.12*	- 0.11
Model 2[b]	0.06*	- 0.13*	- 0.12
**HOMA-β**			
Model 1[a]	2.73*	-4.99*	- 4.65*
Model 2[b]	2.46 (not sig)	- 5.09*	- 4.81*
**HbA1c (%)**			
Model 1[a]	0.02*	- 0.13*	-0.01 (not sig)
Model 2[b]	0.03*	- 0.16*	- 0.01 (not sig)

*P value <0.05

[a]Model 1 adjusted for age (year, continuous), sex, site (A or B), smoking (never-smoker, quitter, current smoker consuming <20 cigarettes/day, or current smoker consuming ≥20 cigarettes/day), alcohol drinking (non-drinker, occasional drinker, or drinker consuming <23 g of ethanol/day, drinker consuming 23-45 g of ethanol/day, or drinker consuming ≥46 g of ethanol/day), work related physical activity (METs-h/day, quartile), leisure-time physical activity (METs-h/week, quartile), night or rotating shift work (yes or no), body mass index (kg/m^2, continuous).

[b] Model 2 additionally adjusted for serum magnesium, serum ferritin, log transformed CRP (mg/dL).
